# A standardized workflow for respiratory‐gated motion management decision‐making

**DOI:** 10.1002/acm2.13705

**Published:** 2022-06-23

**Authors:** Sandra M. Meyers, Kelly Kisling, Todd F. Atwood, Xenia Ray

**Affiliations:** ^1^ Department of Radiation Medicine and Applied Sciences University of California San Diego California USA

**Keywords:** gastrointestinal cancer, lung cancer, motion management, respiratory gating, stereotactic body radiotherapy

## Abstract

**Purpose:**

Motion management of tumors within the lung and abdomen is challenging because it requires balancing tissue sparing with accuracy of hitting the target, while considering treatment delivery efficiency. Physicists can play an important role in analyzing four‐dimensional computed tomography (4DCT) data to recommend the optimal respiratory gating parameters for a patient. The goal of this work was to develop a standardized procedure for making recommendations regarding gating parameters and planning margins for lung and gastrointestinal stereotactic body radiotherapy (SBRT) treatments. In doing so, we hoped to simplify decision‐making and analysis, and provide a tool for troubleshooting complex cases.

**Methods:**

Factors that impact gating decisions and planning target volume (PTV) margins were identified. The gating options included gating on exhale with approximately a 50% duty cycle (Gate3070), exhale gating with a reduced duty cycle (Gate4060), and treating for most of respiration, excluding only extreme inhales and exhales (Gate100). A standard operating procedure was developed, as well as a physics consult document to communicate motion management recommendations to other members of the treatment team. This procedure was implemented clinically for 1 year and results are reported below.

**Results:**

Identified factors that impact motion management included the magnitude of motion observed on 4DCT, the regularity of breathing and quality of 4DCT data, and ability to observe the target on fluoroscopy. These were collated into two decision tables—one specific to lung tumors and another for gastrointestinal tumors—such that a physicist could answer a series of questions to determine the optimal gating and PTV margin. The procedure was used clinically for 252 sites from 213 patients treated with respiratory‐gated SBRT and standardized practice across our 12‐member physics team.

**Conclusion:**

Implementation of a standardized procedure for respiratory gating had a positive impact in our clinic, improving efficiency and ease of 4DCT analysis and standardizing gating decision‐making amongst physicists.

## 1 INTRODUCTION

Radiation is an important treatment modality for many lung^1^ and gastrointestinal (GI) cancers.^2^, ^3^, ^4^ However, motion of these tumors due to respiration necessitates additional consideration to ensure accuracy of radiation delivery. Lung tumors can move over 15 mm superior–inferior and 10 mm in the anterior–posterior and left–right directions, and tumor mobility is dependent on the location within the lung.^5^ Average liver motion measured for a cohort of patients was 13 mm superior–inferior, 2 mm left–right, and 5 mm anterior–posterior,^6^ with similar amplitudes in other abdominal sites. There are numerous strategies for managing motion, which include motion encompassing techniques, respiratory gating, breath‐hold, forced shallow breathing with abdominal compression, and real‐time tumor tracking. Descriptions and comparisons of these methods are widely available in the literature.^7^, ^8^ Motion‐encompassing techniques use an internal target volume (ITV), internal gross tumor volume (IGTV)^9^ and/or larger margins to ensure the tumor is contained within the irradiated volume at any point in the breathing cycle. Because these strategies are relatively simple to implement and delivery is efficient, they are often used for conventionally fractionated treatment. However, stereotactic body radiotherapy (SBRT) is becoming increasingly common for treatment of lung and GI tumors, and presents greater risks due to the high doses per fraction. These treatments benefit from respiratory gating, breath‐hold, abdominal compression, and real‐time tumor tracking techniques, which utilize additional technology or equipment to minimize the amount of normal tissue irradiated.

**FIGURE 1:**
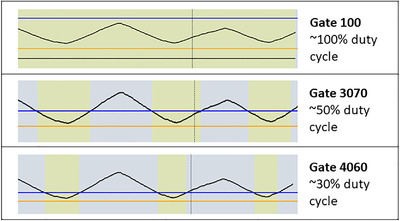
Examples of relatively normal respiratory signals from Varian's Real‐time Position Management^TM^ (RPM) system (Varian Medical Systems, Palo Alto, CA) during treatment delivery, where a high amplitude corresponds to inhale (0% phase), and a low amplitude corresponds to exhale (50% phase). The gating window is defined by the user by adjusting the location of the blue and orange lines, and beam‐on is indicated by yellow. Three potential gating schemes are shown, along with their corresponding gating window: (1 ‐ top) the Gate 100 window is set to encompass all regular phases of breathing motion; (2 ‐ middle) the Gate 3070 window approximately encompasses phases 30% to 70%, and thus the beam is on approximately half the time; (3 ‐ bottom) Gate 4060 encompasses approximately 40% to 60% phases, so the beam is on roughly one third of the time

One of the most common solutions is respiratory gating, which uses a trigger to activate or deactivate radiotherapy delivery. Typically, this means that beam‐on will only occur when the target moves into a specific region (such as the most superior position on exhale), which enables a reduction of the total volume irradiated. Because real‐time monitoring of actual tumor position is not always possible, many systems use a surrogate for tumor motion, such as breathing amplitude. During treatment delivery, the user can select a “gating window,” which will automatically trigger beam‐on when the signal moves within a specified range of amplitudes. Three potential gating window selections are shown in Figure 1. For example, a user may choose to set a wide gating window that encompasses all regular breathing, such that beam‐off is only triggered during an especially large or abnormal inhale or exhale. Alternatively, the user may prefer to deliver treatment when the tumor is in an exhale position, and thus set one of the other two gating windows shown.

**FIGURE 2:**
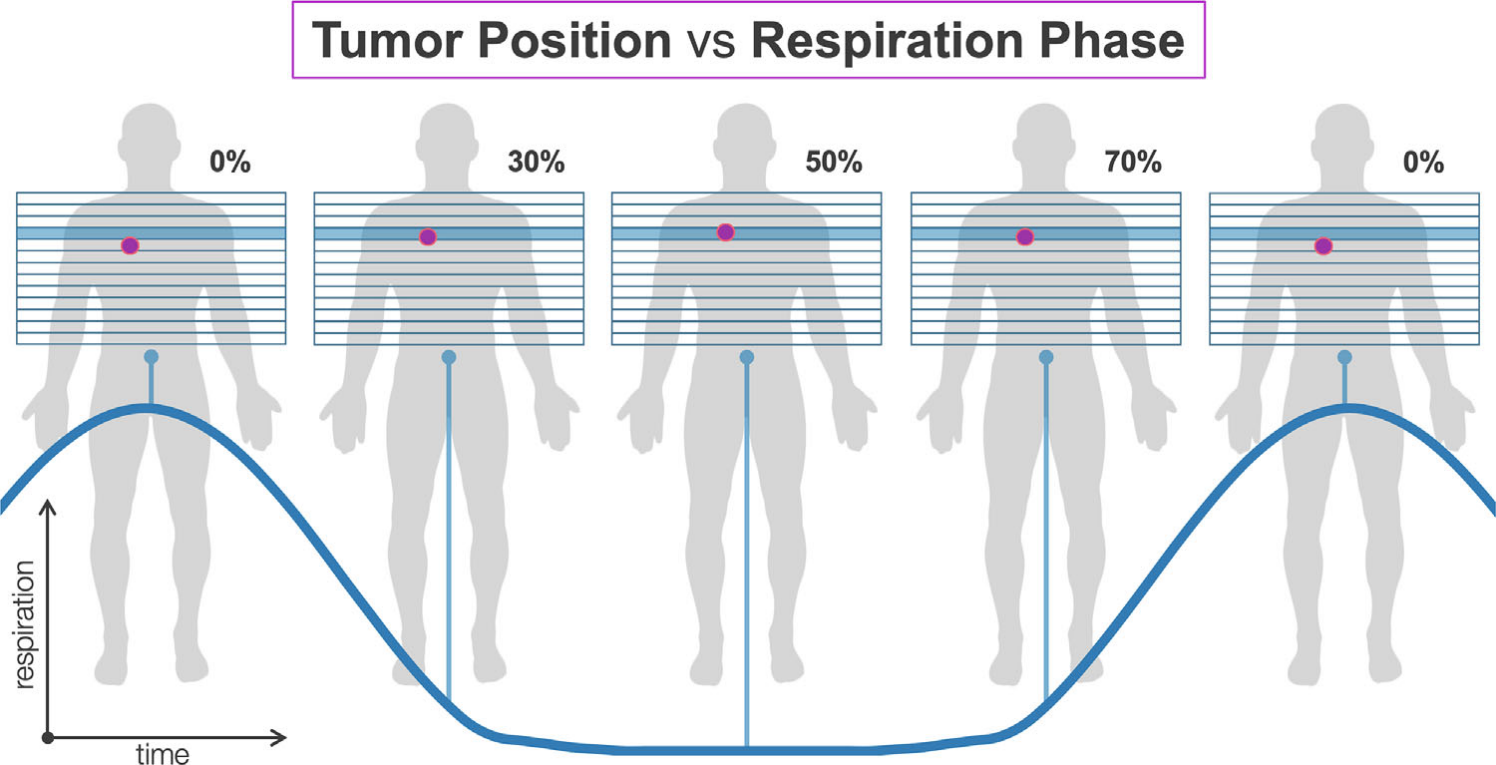
Depiction of a tumor position in each phase of the breathing cycle and corresponding amplitude of the respiratory trace

When using surrogates for respiration, time‐resolved imaging is required to link the signal to internal target motion. This can be achieved with the use of four‐dimensional computed tomography (4DCT) simulation prior to treatment and four‐dimensional cone‐beam CT (4DCBCT) or fluoroscopy during treatment. In 4DCT or 4DCBCT, image data is collected over the course of respiration and sorted into bins based on either breathing amplitude or phase (where 0% phase corresponds to inhale, and 50% to exhale). The result is a series of images (typically 10) that reflect the target position at various points within the breathing cycle (see Figure 2). 4DCT data can inform gating selection for a particular patient, as well as contouring and treatment planning. At treatment, fluoroscopy or 4DCBCT can be beneficial for ensuring setup accuracy, verifying that the external surrogate reflects internal motion, and setting the respiratory gating window.

Although motion management with respiratory gating offers multiple advantages over other strategies, such as allowing patients to breathe freely, improving accessibility, and patient comfort,^10^, ^11^, ^12^ it features challenges where physics expertise can be valuable. For one, gating window selection during treatment delivery must carefully balance reductions in normal tissue irradiation with increased treatment times. In addition, use of time‐resolved imaging, such as 4DCT or fluoroscopy, should be optimized as it increases dose to patients. Further considerations that should guide gating decisions include magnitude of tumor motion, ability to identify target on image guidance, quality of the 4DCT data, and regularity of patient breathing. Medical physicists can play an important role in this analysis, with their knowledge of the technology and theory behind treatment planning and delivery.

While several groups have published on general aspects of respiratory gating, including development of processes, commissioning of equipment,^13^ and guidelines for clinical implementation,^7^, ^14^, ^15^ there are very few resources to guide patient‐specific respiratory gating decision‐making.^16^, ^17^ Large centers, in particular, can benefit from standardized guidelines and workflows to ensure consistency of practice and to reduce errors when multiple hand‐offs are made between team members. The purpose of this work was to develop a standardized approach for making recommendations regarding planning margins and gating decisions for lung and GI SBRT treatment. In doing so, we hoped to simplify decision‐making and analysis, and provide a tool for troubleshooting complex cases. Additionally, we aimed to implement new documentation guidelines to ensure clear communication of a patient's motion management plan to all members involved in the treatment process. This report includes our technique and results after 1 year of clinical implementation.

## 2 METHODS

### 2.1 General clinical workflow

At our clinic, the Real‐time Position Management (RPM) system (Varian Medical Systems Inc., Palo Alto, CA) is used to monitor motion during 4DCT and treatment delivery. Breathing is tracked as the anterior–posterior amplitude of a marker placed on the surface of the abdomen or chest (often referred to as the “respiratory signal”), which is recorded by an infrared camera. Physicists attend all 4DCT simulations, process the imaging data, and analyze the images and motion to provide recommendations for respiratory gating. Images are acquired on our GE Discovery CT 590RT scanner and transferred to MIM Maestro (version 7.0.4, MIM Software Inc., Cleveland, OH), along with the breathing trace, where phase‐based reconstruction over ten respiratory phase bins is automatically performed by the software. The physicist can modify the images by manually selecting the phase bins prior to reconstruction.

Based on the amplitude of the target motion and regularity of the patient's breathing observed on 4DCT, the physicist then makes a gating recommendation to the clinic. Options for the gating recommendation include NoGate, Gate100, Gate3070, and Gate4060 (see Figure 1), which are defined as:

- **NoGate**: used for non‐SBRT treatments. All 4DCT phases are used for ITV contouring. The motion is not monitored at treatment.
- **Gate100**: All 4DCT phases are used for ITV contouring and treatment planning. On treatment, the gating window is set to encompass regular breaths from full exhale to inhale, and large inhales or exhales are excluded.
- **Gate3070**: Only the 30% to 70% phase images (which surround full exhale at 50%) are used for ITV contouring and treatment planning. On treatment, the gating window is set to encompass exhale.
- **Gate4060**: Only the 40% to 60% phase images (which surround full exhale at 50%) are used for ITV contouring and treatment planning. On treatment, the gating window is set to encompass exhale with a shorter duty cycle than Gate3070.

Average intensity projection images are produced for treatment planning and dose calculation, and maximum and/or minimum intensity projections are created for contouring. These projections are generated using only the appropriate phases (e.g., for Gate4060, the 40%, 50%, and 60% phase images). Maximum intensity projections are used for most targets and surrogate (e.g., fiducials), while minimum intensity projections are used for hypo‐intense targets such as liver metastases.

**FIGURE 3:**
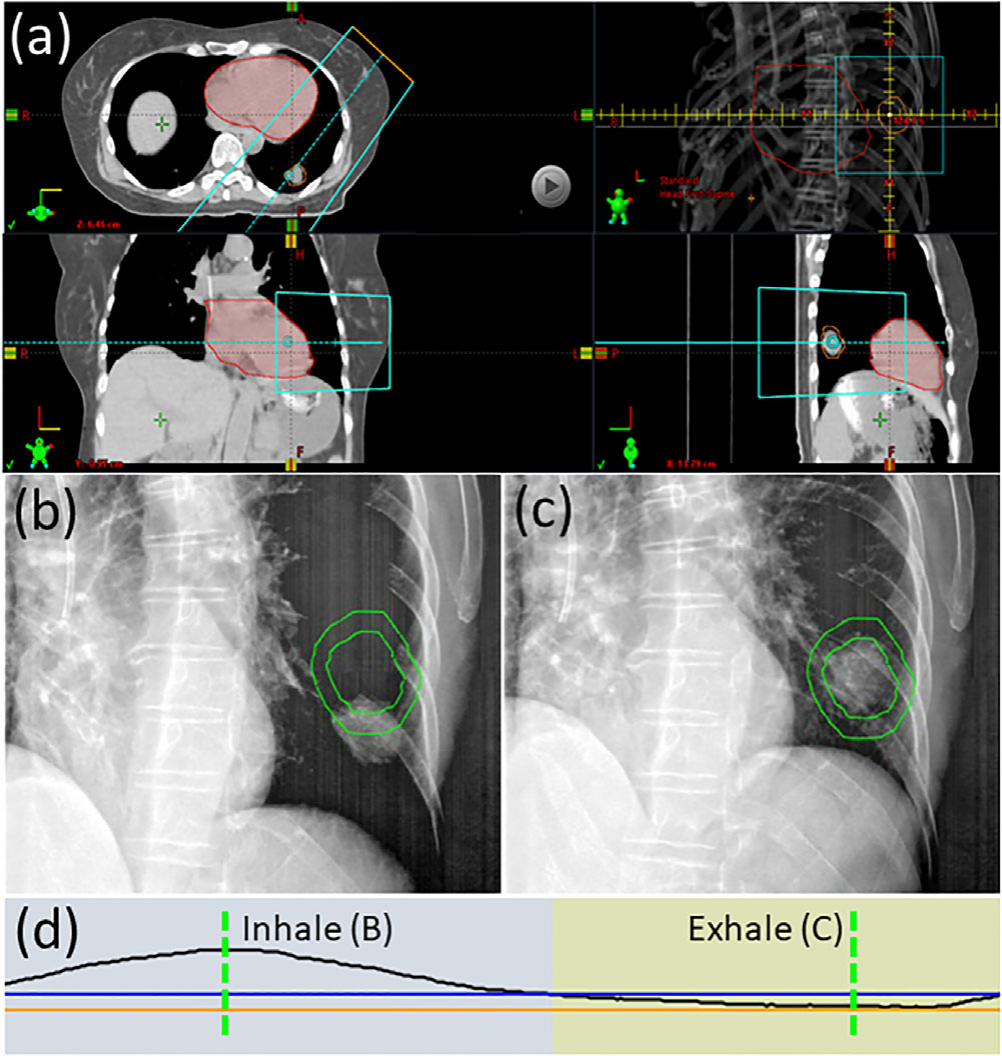
Example of a lung tumor that can be visualized in fluoroscopy with an oblique projection angle. (a) Axial, coronal, and sagittal average CT images, demonstrating that the tumor is positioned posterior to the heart, which will obscure visualization in anterior–posterior projection fluoroscopy. (a) also depicts the beam's eye view of an oblique angled fluoroscopic imaging field, where separation can be observed between the heart (red) and ITV (orange) structures. (b) The ITV and PTV contours (green) and the tumor position on both (b) inhale and (c) exhale oblique fluoroscopy. The corresponding amplitudes of the breathing trace for these images are shown by the dotted green lines in (d). Using fluoroscopy, the gating window (orange and blue lines) is set so that beam‐on (indicated by yellow colorwash) will only occur while the tumor is inside the ITV. This patient is an example of a Gate4060 treatment

Physicists attend all gated treatments. They review CBCT and (when applicable) fluoroscopy imaging to ensure accurate setup and confirm the relationship between the internal target motion and the respiratory trace. Using fluoroscopy, physicists ensure that the tumor or surrogate (e.g., fiducials) does not leave the ITV contour within the gating window specified (see Figure 3c,d). Finally, they monitor the breathing trace throughout treatment to assure treatment is delivered as planned.

### 2.2 Development of a standardized process for motion management recommendation

To standardize motion management analysis and decision‐making, four physicists, including the lung and GI technical site leads, worked together to develop a decision table and documentation template. We began by independently listing all of the factors that affect motion management decision‐making at our clinic. These were then sorted into a logical order to create independent decision tables for lung and GI, respectively. Radiation oncologists with expertise in lung and GI were consulted for clinical insight, particularly on site‐specific planning target volume (PTV) margins and prioritization of tumor coverage and organ sparing. These results were compiled into our Standard Operating Procedure (SOP) document, which included a separate section with the step‐by‐step procedure for generating projection images in MIM, as well as an appendix for trouble‐shooting common difficult scenarios (e.g., changes in breathing at treatment). Finally, a physics consult template document was designed to correspond with the decision tables, which is completed by the physicist performing the 4DCT analysis. It serves as the primary mechanism for documenting and communicating the treatment gating and margin decisions to all members of the team (radiation oncologists, dosimetrists, physicists checking the plan, therapists, and physicists covering the treatment).

The final SOP and physics consult template were sent to all physicists, radiation oncologists, and dosimetrists in our department for comments, before being approved and implemented in the clinic. To train all physicists in the new SOP, each was paired with one of the SOP authors for their first lung and GI case, after its implementation. This allowed them to become competent with the process and have an opportunity for one‐on‐one discussion about changes in the workflow.

### 2.3 Evaluation after 1 year of implementation

After the new process had been implemented for a year, we evaluated the number of patients who had received a 4DCT and the final motion management decisions that were made for each. The use of patient data in this work was approved by our institutional review board (UCSD IRB Project #210663).

## 3 RESULTS

### 3.1 General factors contributing to motion management decision‐making

In developing the standardized gating workflows, we identified the following general factors that contribute to decision‐making for gated treatments:

1. Ability to visualize the target or surrogate (e.g., fiducials) on fluoroscopic imaging
2. Regularity of the breathing and reliability of the 4DCT data at the location of the target
3. The magnitude of target motion

Factor (1) is dependent on the treatment site and location of the target relative to other anatomy. It plays an important role since it determines whether the correlation between the surrogate respiratory signal and actual tumor motion can be verified immediately prior to each treatment fraction. Factor (2) represents whether or not the full extent of target motion over the respiratory cycle can be evaluated from the 4D data. For instance, it is possible that the breathing motion is reduced during the acquisition of the axial image slices containing the tumor, relative to the more common breathing amplitude observed over the course of the 4DCT. As a result, the phase images would not accurately reflect the maximal breathing motion expected on treatment, and thus an ITV generated using these images may not encompass the full motion. If the full inhale to exhale range of motion could not accurately be evaluated, we then determined whether the amplitudes around expiration (e.g., 30%–70% phases) were accurately represented in the 4D data. The amount of motion that could accurately be evaluated (either inhale to exhale or around expiration) was then used to determine Factor (3). For Factor (3) the physicist would review the 4D data and determine the maximal magnitude of target motion in any direction over all phase images (most often between the inhale, or 0%, and exhale, or 50%, phases). If irregular breathing prevented an accurate representation of full motion, they instead measure the motion around exhalation (typically between the 30% or 70% phase and the 50% phase). In some instances, even reliable exhale motion evaluation is not possible, in which case the physicist consults with the site technical lead and the physician.

### 3.2 Motion management recommendations

We then determined a set of recommendation categories that included the gating schemes (described in the Section 2), PTV margin, gating window selection, and use of fluoroscopy at treatment.

Options for PTV margins were determined based on common practice at our institution and discussion with physicians. These include isotropic margins (5 mm for lung and GI, except for 2 mm for pancreas with implanted fiducials) for standard cases, extended margins for increased uncertainty (in which isotropic margins are extended in the superior–inferior direction to 8 mm for lung or 7 mm for GI), or in rare instances other margins at the physicist's discretion. Some examples of when to use extended margins are described in site‐specific sections below.

Options for gating window selection were “Normal” or “Conservative,” and describe how a physicist should set the upper and lower bounds of the gating window on treatment. Typical normal gating windows for each gating scheme are shown in Figure 1, whereas conservative gating windows are narrowed, reducing the duty cycle. We provided specific instructions and example figures in the SOP to ensure all physicists set the gating windows similarly. In general, conservative gating windows are recommended for patients where the correlation between the respiratory trace and actual tumor motion cannot be verified with fluoroscopy.

Fluoroscopy is generally recommended whenever it is possible to visualize the target or surrogate (e.g., fiducials). We also provided recommendations for what structure should be displayed and evaluated during fluoroscopy, which corresponds to an ITV of the tumor or surrogate drawn using the appropriate phase images.

### 3.3 Decision tables and 4D consult documents

**FIGURE 4:**
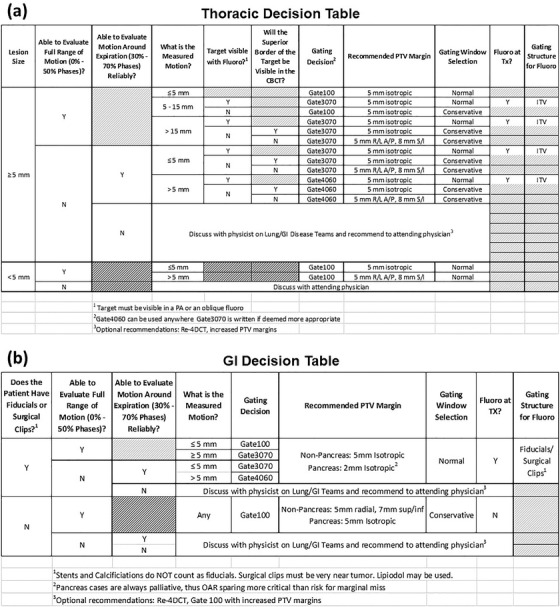
Motion management decision tables for thoracic (a) and GI (b) tumors. N, no; TX, treatment; Y, yes

Finally, we determined how each of the general factors relate to the recommendations, and in doing so constructed both lung and GI‐specific decision tables (see Figure 4). While the tables were intentionally designed for lung and GI tumors (e.g., liver and pancreas), they may also be applied to other thoracic and abdominal sites (e.g., ribs, adrenal glands) with careful consideration. These tables are meant to be used by any physicist analyzing a 4DCT to produce a standardized recommendation for motion management. The first few columns correspond to the general and site‐specific factors that guide the decision‐making process, and the last five columns provide the recommended gating phases, PTV margins, normal versus conservative gating window, use of fluoroscopy, and structure for fluoroscopy. Each column of the table corresponds to an entry in our 4D Physics Consult Document (see Supporting Information), which is included in the patient's chart to communicate recommendations and their justification to all members of the radiotherapy team. Site‐specific considerations for this motion management procedure and decision‐making process are described in the following sections.

### 3.4 Lung‐specific considerations

Our center does not implant fiducials for lung treatments, so the primary consideration for motion management of a lung tumor is whether it will be visible on CBCT and fluoroscopic imaging. Because tumor size impacts visibility on all forms of imaging, it was also deemed a critical factor for decision‐making. From our experience, any tumor with a diameter less than 5 mm will be difficult to visualize on fluoroscopy, and thus Gate100 is recommended. In addition, when these tumors move substantially, motion blurring can cause the target to appear very faint on CBCT; thus, an extended PTV margin is recommended when motion of these small tumors is greater than 5 mm to account for the additional setup uncertainty.

**FIGURE 5:**
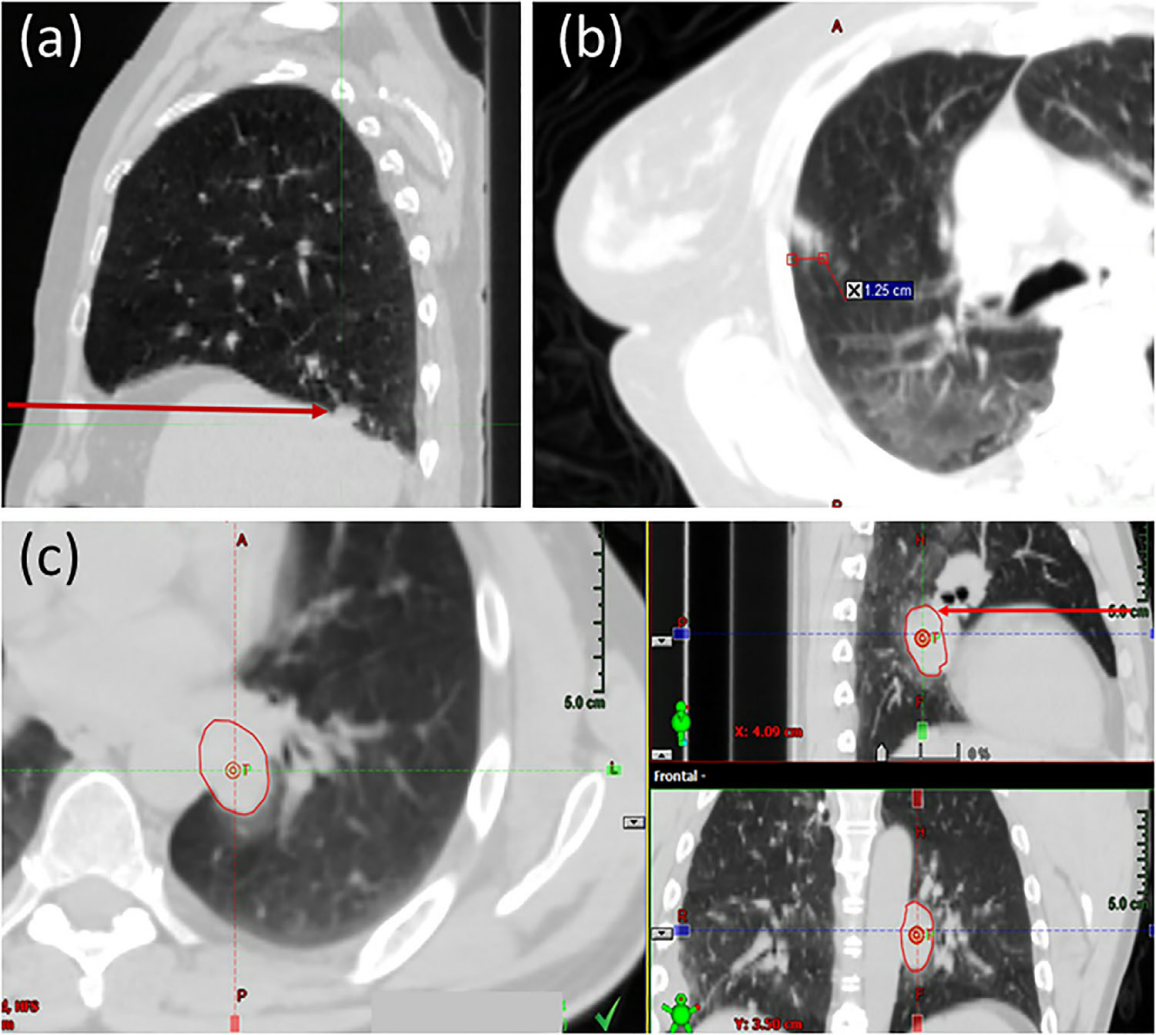
Example cases where thoracic tumors are not visible in fluoroscopic imaging. (a) Sagittal CT image showing tumor located posterior to the diaphragm and inferior to the apex of the diaphragm, such that diaphragm will obscure tumor visualization in an anterior–posterior projection. (b) Axial CT image showing a diffuse tumor, or ground glass object, with signal that is too faint to be observed in fluoroscopy. (c) Axial, sagittal, and coronal CT images of a tumor that is located posterior to mediastinal soft tissue, such as the heart

In our experience, lung tumors cannot be visualized in lateral fluoroscopy because mediastinal tissue and spine tend to dominate the signal in the projection image. Therefore, we recommend the use of an anterior–posterior projection angle for all thoracic fluoroscopic imaging. There are, however, many instances where the tumor may not be visible on an anterior–posterior image either, such as small tumors less than 5 mm in diameter or targets located anterior or posterior to diaphragm, heart or other soft tissue in the mediastinum (see Figure 5). For some of these cases, the tumor may instead be visualized in fluoroscopy with an oblique projection angle (see Figure 3). Thus, a physicist may recommend oblique fluoroscopy on a case‐by‐case basis. Additionally, even when lung tumors can be visualized on fluoroscopy, if they have very little motion from full inhale to exhale (<5 mm), we opted to not recommend the use of fluoroscopy to minimize imaging dose to the patient. In this instance, the physicist will still verify the tumor motion is encompassed by the ITV on CBCT.

When visualization on fluoroscopy is not possible, alignment on the CBCT becomes particularly important. We always recommend aligning to the superior border of the tumor on CBCT^15^, ^18^, ^19^ for all gating schemes since exhale is typically the most reproducible position. If the superior border of the tumor is not visible on CBCT and fluoroscopy is not an option, increases in setup uncertainty could lead to geometric misses when gating on exhale. For these instances, we recommend the use of an extended PTV margin.

### 3.5 GI‐specific considerations

For GI targets, visualization of boundaries can be particularly challenging on daily CBCT and this challenge is further exacerbated by motion. This is also true of other superior abdominal sites, such as retroperitoneal lymph nodes, and so other abdominal sites are also grouped under the “GI” category. Because of these challenges, implanted fiducials must be present for either Gate3070 or Gate4060 to be recommended. Any patients without fiducials or other radiopaque markers (e.g., surgical clips, Lipiodol) that can serve as a surrogate for verifying the gating window are treated using Gate100.

For those patients with fiducials, both posterior‐anterior and lateral fluoroscopy imaging are required at treatment to align the patient and set the gating window. Unlike lung patients, this recommendation also applies to patients being treated during their entire respiratory cycle (Gate100) because the initial setup on CBCT is more challenging. Specifically, it is generally impossible to distinguish the superior border of the target in CBCT. The fiducial contours are used to align the target on CBCT but appear larger than their true size and can create substantial metal artifacts, thus necessitating further confirmation with fluoroscopy. Similar to lung, when fluoroscopy is not used, a conservative gating window at treatment is recommended.

The recommended margins for GI cases vary depending on setup uncertainty. For those cases where fluoroscopy can be used, 2 mm isotropic margins are recommended for pancreas targets and 5 mm isotropic margins are recommended for all other GI targets. When no fiducials are present and thus fluoroscopy is not recommended, extended margins of 5 mm for the pancreas and 5 mm radial, 7 mm superior–inferior for all other GI cases are recommended. In both situations, the margins are smaller for the pancreas because these patients are being treated palliatively and thus organ sparing is prioritized.

### 3.6 Clinical implementation and patient trends from the last year

**FIGURE 6:**
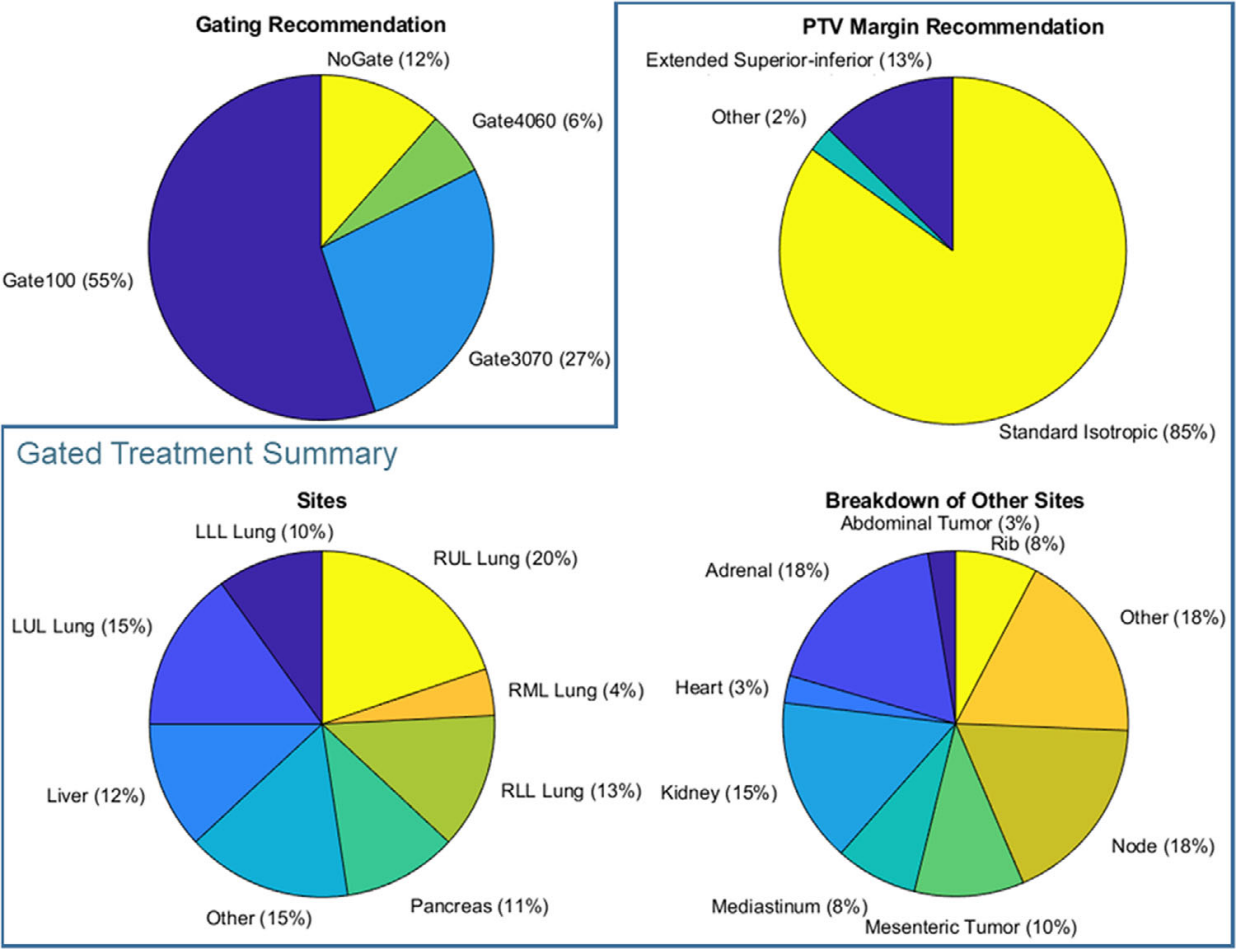
Summary of patients for which physics 4D analysis was performed, where charts inside the blue box summarize SBRT cases that were treated with respiratory‐gated motion management (i.e., excluding “NoGate” cases). Standard isotropic PTV margins are 5 mm for lung and GI, and 2 mm for pancreas with implanted fiducials. Extended superior–inferior margins feature additional superior–inferior expansions on the standard isotropic margins of 7 mm for GI and 8 mm for lung. LLL, left lower lobe; LUL, left lower lobe; RLL, right lower lobe; RML, right medial lobe; RUL, right upper lobe

**FIGURE 7:**
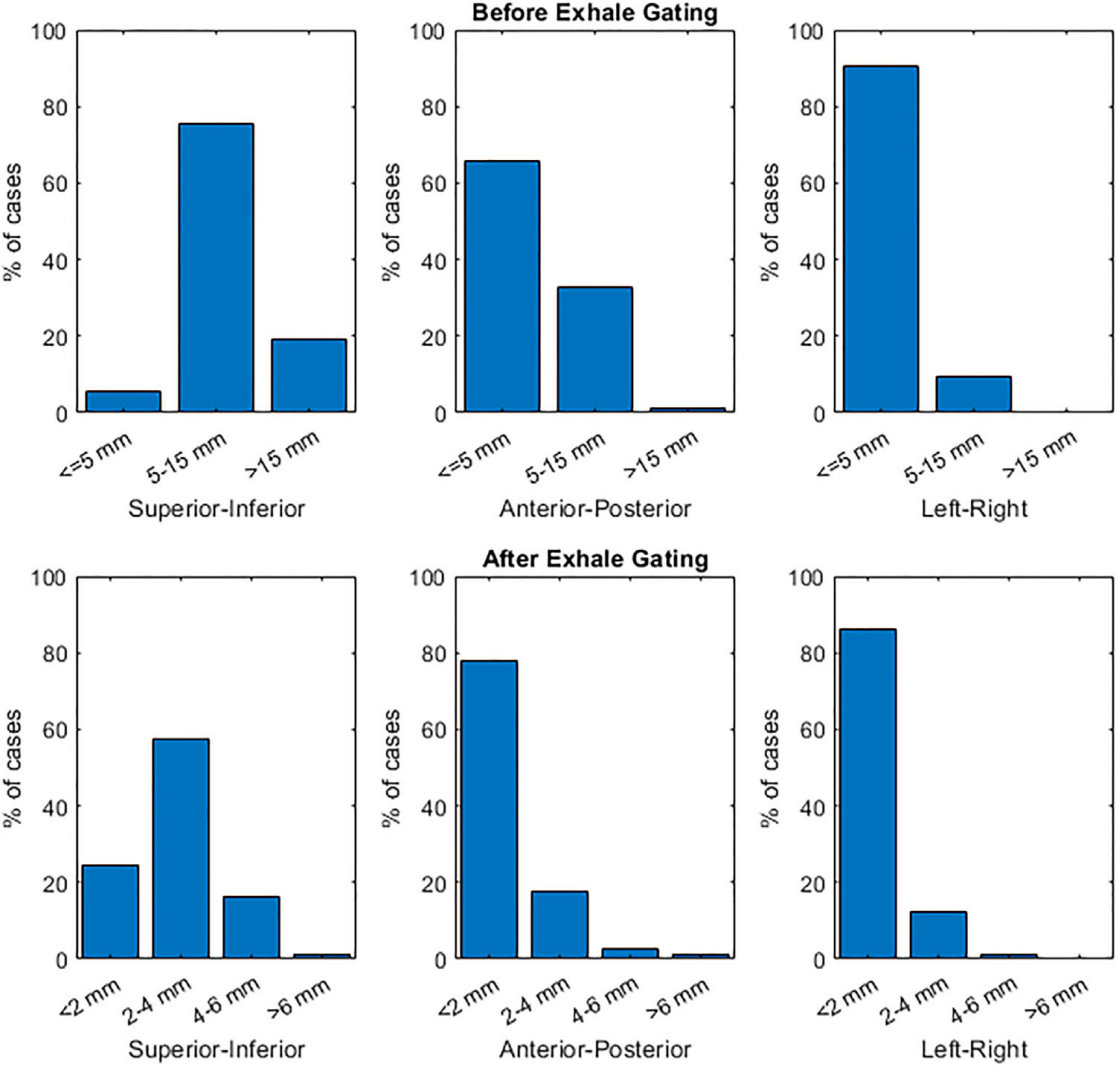
Motion of SBRT treatment targets in all three directions before gating (i.e., during free breathing) and after performing exhale respiratory gating (i.e., Gate3070 or Gate4060), as measured on the 4DCT and reported in the physics 4D consult form

The new motion management procedure was implemented on June 10, 2020. All physicists were fully trained on the procedure within the month following implementation. In the year that followed, 4DCT physics consults were completed for a total 285 treatment sites from 242 patients. Figure 6 displays the distribution of gating recommendations for all sites. 252 sites from 213 patients were treated with respiratory‐gated SBRT. Of these cases, 62% were treated with Gate100, 31% with Gate3070, and 7% with Gate4060. A breakdown of sites and PTV margin recommendations for these cases is shown in Figure 6. 62% of SBRT cases were lung, 12% were liver, and 11% were pancreas tumors, while the remaining 15% corresponded to nodal, adrenal, kidney and other sites. The most common PTV margin recommendation was the standard isotropic margin for that site (5 mm for lung and GI, except 2 mm for pancreas with fiducials). Extended superior–inferior margins were recommended in 13% of cases (which featured an additional superior–inferior expansion of 7 mm for GI and 8 mm for lung). In 2% of cases, physicists recommended other margins due to uncertainty in 4D imaging, irregular breathing, very small lung tumor and/or planned use of a single isocenter for multiple targets, such as 7 mm isotropic (for the latter two reasons). Figure 7 demonstrates the reduction in target motion achieved with exhale gating strategies (Gate3070 or Gate4060), where the largest reductions were in the superior–inferior direction. For the remaining patients that were not gated, the 4DCT served to inform contouring and treatment planning.

## 4 DISCUSSION

The management of breathing motion is challenging because it requires balancing the need for tissue sparing with accuracy of hitting the target, while considering treatment delivery efficiency. Currently, there are few tools available to guide gating and PTV margin selections. Some groups have evaluated the reproducibility of specific population‐based gating windows and parameters^20^, ^21^; however, these studies focused on the magnitude of motion of the external signal at different breathing phases instead of the actual motion of the target, even though the target motion is critical to determine if there is a benefit from gating the treatment. Another group retrospectively measured the residual tumor motion with respiratory‐gated radiotherapy using individualized and population‐based gating window widths^17^ and found that individualizing the window widths decreased the residual motion to acceptable ranges. However, they required data to be collected on‐treatment in order to determine an individual's gating recommendation. Vedam et al. presented a method to determine optimal gating mode (i.e., exhale or inhale gating) and compute patient‐specific PTV margins through automated analysis of diaphragm motion.^16^ While elegant and quantitative, their approach is limited to tumors that are located near the diaphragm and feature similar motion. Our work provides a thorough procedure for determining patient‐specific gating parameters through simple analysis of a patient's 4DCT data. This methodology is flexible as it can be applied to a wide range of tumors located in the thorax and abdomen.

Our primary aim in developing this motion management procedure was to provide clear guidelines on the factors that should be considered and how they should be prioritized when a physicist provides recommendations for respiratory gating. We saw this as a critical need at our clinic where we have 12 physicists; one physicist may provide the initial recommendation for planning while up to 5 different physicists may provide support at each treatment fraction. By standardizing the decision‐making workflow for both lung and GI cancers and improving the documentation of that decision, we hoped to improve confidence in our team members and provide better care for our patients.

An added benefit of the new 4D physics consult documents, SOP, and appendix, is that they can be referenced by other disciplines, such as physicians who need gating information to generate target ITVs, dosimetrists creating the treatment plan, and therapists on treatment. Furthermore, the documents have been useful for both physics and MD resident training. We also noticed greater standardization of recommendations amongst our physicists. Anecdotally, many physicists expressed greater confidence in their gating and motion management‐related recommendations with the new procedure and training. Several physicists commented that the decision tables also helped to reduce the time they spent on 4DCT analysis.

Over a 1‐year timeframe at our clinic, this procedure was used for over 200 patients treated with respiratory‐gated SBRT. The procedure was robust enough to be employed in a multitude of sites that spanned from standard lung and GI sites to heart, kidneys, ribs, and lymph nodes, among others. About two thirds of gated cases employed a Gate100 gating scheme, with the remainder featuring exhale gating. While Gate100 irradiates a larger amount of tissue, it reduces the chance of target miss and therefore was deemed the safer option for many cases that featured challenges with target localization on imaging, inability to verify with fluoroscopy, or irregular breathing. For cases without these challenges and with relatively large target motion, exhale gating was preferred. The majority of tumors moved 5–15 mm in the superior–inferior direction, and this motion was most often reduced to 2–4 mm with exhale gating. Our clinic has opted to gate on exhale (as opposed to inhale) because breathing movement is more reproducible, residual tumor motion is minimal, and the duty cycle can be increased. However, it should be noted that the reduction in lung volume on exhale can increase relative dose‐volume metrics like V20.

The PTV margin options were based on prior experience at our clinic and discussion with physicians. While most cases employed the standard PTV margins, our analysis method helped to identify a subset of patients (∼15%) that could benefit from increasing margins by 2–5 mm to account for uncertainties in imaging and other factors. For instance, if a full inhale breath was not taken during acquisition of the axial slices containing the tumor in the 4DCT, an artificially small ITV could result, potentially leading to geometric miss. By having physicists critically review all cases and increasing the margins or recommending appropriate exhale gating strategies, this risk is reduced. This procedure could be further improved by computing patient‐specific PTV margins.^16^

There are some limitations to our methodology. For one, the procedure cannot account for every possible challenge encountered during 4DCT or on‐treatment. However, we believe that by providing an order in which to consider the effect of the most common challenges (irregular breathing, large breathing motion, and differences between treatment sites), the physicist can make a guided decision for the vast majority of cases, and can more easily recognize when they are encountering an unusual patient. For those difficult cases, physicists are encouraged to consult with other physics and physician colleagues to make the best decision. Our appendix provides additional guidance for many of the challenges that can be encountered.

Our motion management treatments rely on CBCT and fluoroscopy for image guidance; however, the use of 4DCBCT could improve the visibility of targets at the time of treatment, particularly for GI targets with a large amount of motion. On the other hand, 4DCBCT also increases treatment time, doubles the imaging dose,^22^ and suffers from reduced image quality with irregular breathing and aliasing artifacts due to the limited number of projections for each phase.^23^ If 4DCBCT was employed, the current decision‐making templates may need to be revised to account for the increased certainty in target position with breathing amplitude.

Finally, the procedure is reliant on physicists attending all 4DCT and gated procedures, and spending anywhere from 5 min to an hour analyzing each 4DCT in MIM. Some clinics may not have the resources to allocate this much physics time to these activities. One option is to train simulation CT therapists to cover 4DCTs alone. Based on our experience, physicists are the discipline with the expertise in the technology, limitations, and implications of gating decisions, so we believe physicists should minimally perform the 4DCT analysis and attend every treatment. For clinics where this is not feasible, alternative motion management strategies may be more appropriate.

While this procedure is only directly applicable within the context of a gating system that uses an external respiratory signal paired with fluoroscopic imaging capabilities, the framework could be adapted to other motion management solutions, such as abdominal compression, active breathing control, and real‐time tumor tracking. The list of considerations presented could be a useful starting point for clinics who want to re‐evaluate their use of one of these other technologies. Similarly, our decision‐making grids could be easily modified for their needs by removing or adding the factors that affect their gating strategy, margins, or use of image guidance.

## 5 CONCLUSION

We developed a standardized approach for motion management of lung and GI SBRT treatments using respiratory gating. This consisted of a standard operating procedure that guides physics analysis of 4DCT data, gating and PTV margin recommendations, and on‐treatment decision‐making. A standardized 4D physics consult document was developed to provide clear communication of a patient's customized motion management plan to all members of the radiation oncology team. After training, the procedure was implemented clinically and results were reported here following 1 year of implementation. The approach was applicable to numerous treatment sites and facilitated clear communication between team members and disciplines throughout the treatment process. Additionally, it standardized motion management decision‐making amongst our large physics team and improved ease of 4DCT analysis and gating recommendation. We believe procedures similar to this one would be valuable in any clinic employing motion management, and that physicists play a crucial role in guiding the optimal and safe use of this technology.

## CONFLICT OF INTEREST

Kelly Kisling, Todd Atwood, and Xenia Ray acknowledge Honoraria and speaker fees from Varian Medical Systems. Xenia Ray has a research agreement with Varian Medical Systems.

## AUTHOR CONTRIBUTIONS

All authors contributed to the conception of the work, acquisition, analysis, and interpretation of the data; drafted the work; approved the published version; and agree to be accountable for all aspects of the work.

## Supporting information

Supporting Information
The below documents consist of the text and drop‐down options used to generate our 4D consult template, and an example filled consult for a patient with a lung tumor treated with Gate4060. RUL = right upper lobe, RML = right medial lobe, RLL = right lower lobe, LUL = left lower lobe, LLL = left lower lobe, MIP = maximum intensity projection, MIN = minimum intensity projection, EBHC = exhale breath‐hold with contrast, AVG = average.

## References

[1] Prezzano KM , Ma SJ , Hermann GM , Rivers CI , Gomez‐Suescun JA , Singh AK . Stereotactic body radiation therapy for non‐small cell lung cancer: a review. World J Clin Oncol. 2019;10(1):14‐27. 10.5306/wjco.v10.i1.14 30627522 PMC6318482

[2] Bockbrader M , Kim E . Role of intensity‐modulated radiation therapy in gastrointestinal cancer. Expert Rev Anticancer Ther. 2009;9(5):637‐647. 10.1586/era.09.16 19445580

[3] Rusthoven KE , Kavanagh BD , Cardenes H , et al. Multi‐institutional phase I/II trial of stereotactic body radiation therapy for liver metastases. J Clin Oncol Off J Am Soc Clin Oncol. 2009;27(10):1572‐1578. 10.1200/JCO.2008.19.6329 19255321

[4] Bujold A , Massey CA , Kim JJ , et al. Sequential phase I and II trials of stereotactic body radiotherapy for locally advanced hepatocellular carcinoma. J Clin Oncol Off J Am Soc Clin Oncol. 2013;31(13):1631‐1639. 10.1200/JCO.2012.44.1659 23547075

[5] Liu HH , Balter P , Tutt T , et al. Assessing respiration‐induced tumor motion and internal target volume using four‐dimensional computed tomography for radiotherapy of lung cancer. Int J Radiat Oncol Biol Phys. 2007;68(2):531‐540. 10.1016/j.ijrobp.2006.12.066 17398035

[6] Brandner ED , Wu A , Chen H , et al. Abdominal organ motion measured using 4D CT. Int J Radiat Oncol Biol Phys. 2006;65(2):554‐560. 10.1016/j.ijrobp.2005.12.042 16690437

[7] Keall PJ , Mageras GS , Balter JM , et al. The management of respiratory motion in radiation oncology report of AAPM Task Group 76. Med Phys. 2006;33(10):3874‐3900. 10.1118/1.2349696 17089851

[8] Molitoris JK , Diwanji T , Snider JW , et al. Advances in the use of motion management and image guidance in radiation therapy treatment for lung cancer. J Thorac Dis. 2018;10(Suppl 21):S2437‐S2450. doi:10.21037/jtd.2018.01.155 30206490 PMC6123191

[9] Chang JY , Dong L , Liu H , et al. Image‐guided radiation therapy for non‐small cell lung cancer. J Thorac Oncol. 2008;3(2):177‐186. 10.1097/JTO.0b013e3181622bdd 18303441

[10] Burnett SSC , Sixel KE , Cheung PCF , Hoisak JDP . A study of tumor motion management in the conformal radiotherapy of lung cancer. Radiother Oncol. 2008;86(1):77‐85. 10.1016/j.radonc.2007.11.017 18077031

[11] Giraud P , Morvan E , Claude L , et al. Respiratory gating techniques for optimization of lung cancer radiotherapy. J Thorac Oncol Off Publ Int Assoc Study Lung Cancer. 2011;6(12):2058‐2068. 10.1097/JTO.0b013e3182307ec2 22052228

[12] Bissonnette JP , Franks KN , Purdie TG , et al. Quantifying interfraction and intrafraction tumor motion in lung stereotactic body radiotherapy using respiration‐correlated cone beam computed tomography. Int J Radiat Oncol. 2009;75(3):688‐695. 10.1016/j.ijrobp.2008.11.066 19395200

[13] Hickling SV , Veres AJ , Moseley DJ , Grams MP . Implementation of free breathing respiratory amplitude‐gated treatments. J Appl Clin Med Phys. 22(6):119‐129. 10.1002/acm2.13253 PMC8200514 33982875

[14] Matsuo Y , Onishi H , Nakagawa K , et al. Guidelines for respiratory motion management in radiation therapy. J Radiat Res. 2013;54(3):561‐568. 10.1093/jrr/rrs122 23239175 PMC3650747

[15] Brandner ED , Chetty IJ , Giaddui TG , Xiao Y , Huq MS . Motion management strategies and technical issues associated with stereotactic body radiotherapy of thoracic and upper abdominal tumors: a review from NRG oncology. Med Phys. 2017;44(6):2595‐2612. 10.1002/mp.12227 28317123 PMC5473359

[16] Vedam SS , Keall PJ , Kini VR , Mohan R . Determining parameters for respiration‐gated radiotherapy. Med Phys. 2001;28(10):2139‐2146. 10.1118/1.1406524 11695776

[17] Fuji H , Asada Y , Numano M , et al. Residual motion and duty time in respiratory gating radiotherapy using individualized or population‐based windows. Int J Radiat Oncol. 2009;75(2):564‐570. 10.1016/j.ijrobp.2009.03.074 19735882

[18] Glide‐Hurst CK , Chetty IJ . Improving radiotherapy planning, delivery accuracy, and normal tissue sparing using cutting edge technologies. J Thorac Dis. 2014;6(4):303‐318. 10.3978/j.issn.2072-1439.2013.11.10 24688775 PMC3968554

[19] Tai A , Christensen JD , Gore E , Khamene A , Boettger T , Li XA . Gated treatment delivery verification with on‐line megavoltage fluoroscopy. Int J Radiat Oncol. 2010;76(5):1592‐1598. 10.1016/j.ijrobp.2009.08.005 20133069

[20] Oh SA , Yea JW , Kim SK . Statistical determination of the gating windows for respiratory‐gated radiotherapy using a visible guiding system. PLOS ONE. 2016;11(5):e0156357. 10.1371/journal.pone.0156357 27228097 PMC4881953

[21] Oh SA , Yea JW , Kim SK , Park JW . Optimal gating window for respiratory‐gated radiotherapy with real‐time position management and respiration guiding system for liver cancer treatment. Sci Rep. 2019;9(1):4384. 10.1038/s41598-019-40858-2 30867519 PMC6416406

[22] Varian Medical Systems , Palo Alto, CA, USA. TrueBeam Technical Reference Guide ‐ Volume 2: Imaging. Publication ID: P1033684‐001‐A; 2021. 213 p.

[23] Sonke JJ , Zijp L , Remeijer P , van Herk M . Respiratory correlated cone beam CT. Med Phys. 2005;32(4):1176‐1186. 10.1118/1.1869074 15895601

