# Supplementary material for: A standardized workflow for respiratory‐gated motion management decision‐making
Source: J Appl Clin Med Phys. 2022 Jun 23;23(8):e13705. doi: 10.1002/acm2.13705 (PMC9359043; doi:10.1002/acm2.13705)

XXXX Cancer Center
Radiation Oncology
Special Physics Consult
4D CT Sim

Patient Name:
MRN:
Date of Service:
Requested by: [MD Name]

A member of the physics faculty from the Department of Radiation Oncology was asked to consult on the optimal radiation therapy treatment approach due to respiration-induced motion in the treatment area. The evaluation and recommendations are listed below.

A 4D CT simulation using the RPM system was performed to determine the range of motion of the tumor. The patient's breathing pattern was carefully monitored, and imaging parameters were determined to maximize image quality for tumor delineation and radiation therapy treatment planning.

Site (drop down): RUL lung; RLL lung; RML lung; LUL lung; LLL lung; Liver; Pancreas; Other (see details below)
Additional information: free text

Recommendations:

<u>Gating Technique</u> (drop-down): Gate100; Gate3070; Gate4060; NoGate

<u>Treatment Planning</u>

PTV margins (drop-down): 5 mm Isotropic (Lung/GI Standard); 5 mm Radial, 8 mm S/I (Lung Extended); 5 mm Radial, 7 mm S/I (GI Extended); 2 mm Isotropic (Pancreas with Fiducials); 5 mm Isotropic (Pancreas without Fiducials); Other (see details below)
Additional information: free text

Images for ITV delineation:

Phases	MIP	MIN	EBHC
❑ 0%/50%<br>❑ 30%/50%/70%<br>❑ 40%/50%/60%<br>❑ None	❑ MIP100<br>❑ MIP3070<br>❑ MIP4060<br>❑ None	❑ MIN100<br>❑ MIN3070<br>❑ MIN4060<br>❑ None	❑ EBHC<br>❑ None

Additional information: free text

Images for treatment planning/dose calculation (drop-down): AVG100; AVG3070; AVG4060

Treatment Delivery

Fluoro at treatment (drop-down): None; AP/PA; AP/PA and Lateral
Gating structure for fluoro (drop-down): NA; ITV; Fiducials; Surgical Clips; Other (see details below)
Images for contouring surrogate gating structure (drop-down): NA; 0/50% phases and MIP100; 30/50/70% phases and MIP3070; 40/50/60% phases and MIP4060; Other (see details below)
Gating window selection (drop-down): Normal; Conservative

Additional information: free text

Justification:

Structure that was assessed (drop-down): Target; Fiducials; Surgical Clips; Other (see details below)
Additional information: free text

Motion was reliably evaluated over phases (drop-down): 0-50%; 30-70%; 40-60%; Could not be reliably evaluated (see details below)
Additional information: free text

Measured motion over the above phases:

- S/I (drop-down): ≤5 mm; 5-15 mm; >15 mm
- A/P (drop-down): ≤5 mm; 5-15 mm; >15 mm
- L/R (drop-down): ≤5 mm; 5-15 mm; >15 mm

Selected gating will reduce motion to:

- S/I (drop-down): No change (Gate100/NoGate); <2 mm; 2-4 mm; 4-6mm; >6 mm
- A/P (drop-down): No change (Gate100/NoGate); <2 mm; 2-4 mm; 4-6mm; >6 mm
- L/R (drop-down): No change (Gate100/NoGate); <2 mm; 2-4 mm; 4-6mm; >6 mm

Additional comments: free text

Screenshot of anatomy that was assessed, and breathing trace:
(include coronal and sagittal view and breathing trace of slab containing tumor/fiducial)

XXXX Cancer Center
Radiation Oncology
Special Physics Consult
4D CT Sim

Patient Name: JOHN DOE
MRN: 12345678
Date of Service: 3/1/2021
Requested by: Jane Smith

A member of the physics faculty from the Department of Radiation Oncology was asked to consult on the optimal radiation therapy treatment approach due to respiration-induced motion in the treatment area. The evaluation and recommendations are listed below.

A 4D CT simulation using the RPM system was performed to determine the range of motion of the tumor. The patient's breathing pattern was carefully monitored, and imaging parameters were determined to maximize image quality for tumor delineation and radiation therapy treatment planning.

Site: LLL Lung
Additional information:

Recommendations:

Gating Technique: Gate4060

Treatment Planning

PTV margins: 5 mm Radial, 8 mm S/I (Lung Extended)
Additional information: Suspect difficulty observing tumor clearly on CBCT due to large amount of motion sup-inf and small size, so recommend extended margin

Images for ITV delineation:

Phases	MIP	MIN	EBHC
☐ 0%/50%<br>☐ 30%/50%/70%<br>☒ 40%/50%/60%<br>☐ None	☐ MIP100<br>☐ MIP3070<br>☐ MIP4060<br>☒ None	☐ MIN100<br>☐ MIN3070<br>☐ MIN4060<br>☒ None	☐ EBHC<br>☒ None

Additional information: Must contour using individual phase images as diaphragm will obscur tumor in MIP.

Images for treatment planning/dose calculation: AVG4060

<u>Treatment Delivery</u>

Fluoro at treatment: None
Gating structure for fluoro: NA
Images for contouring surrogate gating structure: NA
Gating window selection: Conservative

Additional information: Tumor will not be visible on fluoro due to small size and position behind diaphragm.

Justification:

Structure that was assessed: Target
Additional information:

Motion was reliably evaluated over phases: 0-50%
Additional information:

Measured motion over the above phases:

- S/I   >15 mm
- A/P   ≤5 mm
- L/R   ≤5 mm

Selected gating will reduce motion to:

- S/I: 4-6 mm
- A/P: <2 mm
- L/R: <2 mm

Additional comments:

Screenshot of anatomy that was assessed, and breathing trace:

(Include coronal and sagittal view and breathing trace of slab containing tumor/fiducial)

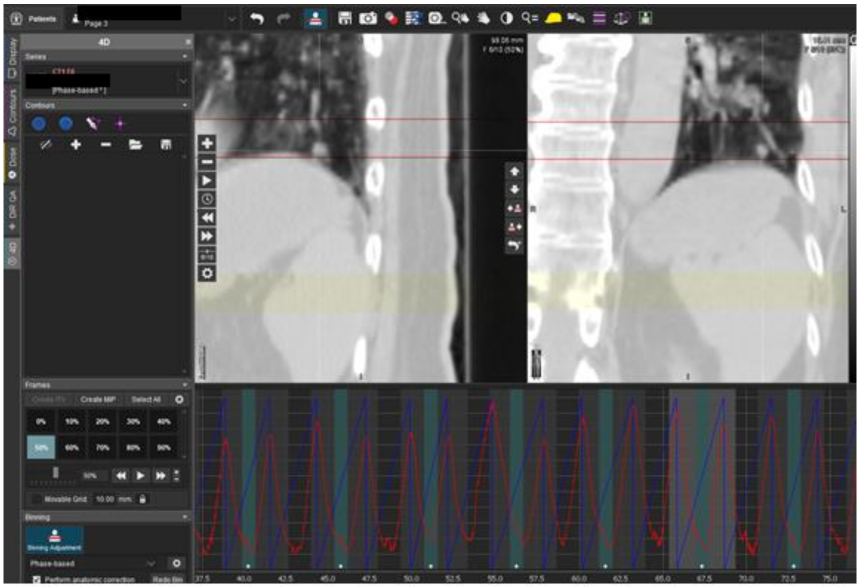

Supplement: Supplementary file 1 — Supporting Information The below documents consist of the text and drop‐down options used to generate our 4D consult template, and an example filled consult for a patient with a lung tumor treated with Gate4060. RUL = right upper lobe, RML = right medial lobe, RLL = right lower lobe, LUL = left lower lobe, LLL = left lower lobe, MIP = maximum intensity projection, MIN = minimum intensity projection, EBHC = exhale breath‐hold with contrast, AVG = average. [file ACM2-23-e13705-s001.pdf]
